# RE: George *et al.* ‘High seroprevalence of COVID-19 infection in a large slum in South India; what does it tell us about managing a pandemic and beyond?’

**DOI:** 10.1017/S0950268821001370

**Published:** 2021-06-28

**Authors:** Victor M. Cardenas

**Affiliations:** Department of Epidemiology, Fay W. Boozman College of Public Health University of Arkansas for Medical Sciences, 4301 W. Markham #820, Little Rock, 72205, Arkansas, USA

Sir, I read with interest the recent paper by George *et al.* [1], which reported a high prevalence of severe acute respiratory syndrome-coronavirus-2 (SARS-CoV-2) antibody in a random sample of residents of DJ Halli, Bengaluru, Karnataka, India. The authors concluded that ‘[the] “high seroprevalence” suggests that the worst [was] over. It [was] a relief to realise that the virus spared most lives in this slum. With more than half of the population already being infected, the infection curve [was] likely to have started its journey down and coronavirus disease-2019 (COVID-19) will cease to be a public health problem in DJ Halli’. The prediction of the authors was accurate. However, I could not find in the paper the dates when data were collected; one can only assume the survey took place around October 2020. The first wave seemed to have affected that particular slum, but I am uncertain it affected at the same time other deprived neighbourhoods. A contemporaneous survey in the entire State of Karnataka which found a prevalence of SARS-CoV-2 infection at 16%, is quoted by George et al. as follows ‘the survey did not include population from slums’ to explain the overall lower 16% prevalence of past infection in the State. The second wave of COVID-19 in early 2021 may not spare Bengaluru's other slums, but it is noticeable that DJ Halli is mentioned as one of the wards with fewer reported cases, while other slums such as those in the Mahadevapura zone experienced a high risk [2]. The spread of COVID-19 may be more complex, with many determinants interacting in place, time and person, one of which is the lack of adequate housing, recently reaffirmed as a ‘component of the right to an adequate standard of living’ by the United Nations [3].
